# Maternal BLV Infection Markers and *BoLA-DRB3* Polymorphism Are Associated with BLV Infection Outcomes in Dairy Youngstock

**DOI:** 10.3390/ijms27198627

**Published:** 2026-09-26

**Authors:** Madison E. Sokacz, Aneta Pluta, Bo Norby, Paul Bartlett, Kelly R. B. Sporer, Casey Droscha, Tasia M. Taxis

**Affiliations:** 1 Department of Animal Science, Michigan State University, East Lansing, MI 48824, USA; sokaczma@msu.edu; 2 Department of Virology and Viral Animal Diseases, National Veterinary Research Institute, 24-100 Puławy, Poland; 3 College of Veterinary Medicine, Michigan State University, East Lansing, MI 48824, USA; 4 Cornerstone Ag Management, LLC, Hastings, MI 49058, USA; 5 CentralStar Cooperative Inc., Lansing, MI 48910, USA

**Keywords:** bovine leukemia virus (BLV), proviral load, youngstock, maternal infection markers, *BoLA-DRB3* polymorphism, epitope breadth, host genetics, longitudinal qPCR

## Abstract

Bovine leukemia virus (BLV) is a deltaretrovirus that establishes lifelong infection in cattle and remains highly prevalent in dairy herds. This study evaluated associations between maternal BLV infection markers, daughter *BoLA-DRB3* variation, and BLV outcomes in dairy youngstock. We studied 201 dam–daughter pairs from five dairy farms. BLV status was assessed using ELISA and quantitative PCR (qPCR); maternal qPCR results were available for 126 dams, and daughters were sampled longitudinally during the youngstock period. *BoLA-DRB3* genotypes were determined in 199 daughters, and genotype-level epitope breadth was defined as the non-redundant union of predicted BLV Gag epitopes across both alleles. BLV proviral DNA was detected in 33 of 200 daughters (16.5%) in the qPCR outcome used for the main analysis. Among 38 daughters with BLV proviral DNA detected at least once, first detection occurred preweaning in 2, prebreeding in 24, and post-breeding in 12. Maternal ELISA positivity was associated with higher odds of daughter BLV proviral DNA detection (OR 2.74, 95% CI 1.10–6.83), whereas maternal PVL showed a positive but imprecise association (OR 1.94, 95% CI 0.89–4.22). Greater predicted epitope breadth was nominally associated with lower odds of BLV proviral DNA detection (OR 0.76, 95% CI 0.60–0.97; q = 0.162) and BLV seropositivity (OR 0.80, 95% CI 0.65–0.98; q = 0.162), but neither association remained significant after correction for multiple testing. BoLA-DRB3*014:01:01 carriers also had lower observed frequencies of BLV proviral DNA detection and BLV seropositivity. Overall, maternal infection markers were associated with later BLV outcomes in daughters, whereas the *BoLA-DRB3* findings provide suggestive evidence of host immunogenetic heterogeneity.

## 1. Introduction

Bovine leukemia virus (BLV) is a deltaretrovirus that establishes lifelong infection through reverse transcription and integration of viral DNA into the host genome. BLV is endemic in the United States, and previous national studies detected infected animals in 89% of U.S. dairy herds [1,2]. Most infected cattle remain clinically asymptomatic, but BLV has been associated with immune dysregulation, increased susceptibility to secondary disease, reduced productivity, premature culling, and economic loss [2,3,4]. The infection can therefore remain unrecognized within individual herds when routine testing is not incorporated into herd-health programs. In the United States, BLV is not included among the current federal cattle disease eradication programs, and national epidemiological estimates have largely been generated through periodic surveillance rather than universal compulsory herd-level testing [2].

BLV transmission requires transfer of infected lymphocytes. Horizontal transmission can occur through blood-contaminated management procedures and biting insects [5,6,7,8,9,10]. Maternal BLV infection may influence early-life exposure through in utero transmission or exposure to infected lymphocytes in colostrum and milk, while horizontal transmission during rearing provides an additional source of infection [11,12,13,14,15,16,17]. Evaluating maternal infection markers in relation to daughter outcomes is therefore relevant for identifying epidemiologic risk patterns and for understanding when infection becomes detectable during the youngstock period.

BLV infection is commonly assessed by serological and molecular methods. ELISA detects anti-BLV antibodies, whereas quantitative PCR (qPCR) detects proviral DNA and can quantify proviral load (PVL) [18,19,20,21]. PVL is best interpreted as a relative marker of infection burden and transmission potential rather than a binary indicator of epidemiological relevance. Animals with higher PVL generally represent higher transmission risk, whereas low or currently undetectable PVL should not be interpreted as absence of infection risk, particularly when the herd-level objective is elimination rather than surveillance or stepwise prevalence reduction [22,23,24].

Host genetic variation may also contribute to BLV phenotypes. The highly polymorphic bovine MHC class II locus *BoLA-DRB3* encodes a molecule involved in presentation of viral peptides to CD4+ T lymphocytes, and *BoLA-DRB3* alleles have been associated with differences in BLV susceptibility and PVL [16,21,25,26,27,28,29]. Immunoinformatic analyses have further identified substantial allele-specific variation in predicted binding of BLV Gag CD4+ T-cell epitopes [30]. Because the two alleles carried by an animal can bind overlapping or distinct epitopes, a genotype-level measure based on the non-redundant union of predicted epitopes more directly captures predicted repertoire breadth than the arithmetic mean of allele-specific counts. BoLA-DRB3*014:01:01 is particularly relevant because independent studies in Holstein cattle have repeatedly associated this allele with lower PVL or resistance-associated BLV phenotypes [29,31].

The objectives of this study were therefore to (i) quantify associations between maternal BLV infection markers and daughter BLV outcomes while accounting for daughter age, farm, sampling interval, and daughter *BoLA-DRB3* epitope breadth; (ii) describe longitudinal detection of BLV proviral DNA by qPCR during the youngstock period; and (iii) evaluate associations between daughter *BoLA-DRB3* genotype-level predicted epitope breadth and BLV proviral DNA detection, PVL magnitude, and ELISA positivity for anti-BLV antibodies.

## 2. Results

### 2.1. BLV Infection Status, Age at Sampling, and Longitudinal qPCR Detection in Daughters

A total of 201 dam-daughter pairs from five dairy farms were included in the analysis from a larger longitudinal cohort of 254 youngstock. The qPCR outcome used for the main analysis was available for 200 daughters: BLV proviral DNA was detected in 33 daughters (16.5%) and was not detected in 167; one daughter had no later-youngstock qPCR result. Among these outcomes, 194 were based on post-breeding samples and 6 on prebreeding samples because those daughters were unavailable for post-breeding sampling following culling. The median age at the qPCR outcome was 498 days (interquartile range [IQR]: 473–525.3; range 344–606; *n* = 200). Farm-specific median ages were 488.5 days on Farm 1 (*n* = 38; range 443–602), 531 days on Farm 2 (*n* = 57; range 500–586), 473 days on Farm 3 (*n* = 16; range 350–515), 467 days on Farm 4 (*n* = 45; range 344–555), and 489.5 days on Farm 5 (*n* = 44; range 354–606) (Supplementary Table S2). Daughter ELISA results were available for 200 animals, of which 43 (21.5%) were seropositive for BLV and 157 were seronegative. The median age at ELISA testing was 497.5 days (IQR 472–524.3; range 335–606). No ELISA sample was collected at ≤200 days of age; therefore, a sensitivity analysis excluding ages at which passively acquired colostral antibodies would be expected to persist removed no observations.

Longitudinal BLV qPCR results were available at up to three sampling stages. Among daughters with an available qPCR result, BLV proviral DNA was detected in 2 of 196 at preweaning, 26 of 179 at prebreeding, and 32 of 194 at post-breeding. BLV proviral DNA was detected at least once in 38 daughters. First detection occurred preweaning in 2 animals (at 2 and 7 days of age), prebreeding in 24 animals (median age 377 days; range 335–450), and post-breeding in 12 animals (median age 517.5 days; range 471–586). Of the 36 daughters first detected at prebreeding or post-breeding, 34 had at least one earlier qPCR result in which BLV proviral DNA was not detected.

Maternal milk ELISA testing preceded daughter birth by a median of 275 days (range 5–442 days) and preceded the daughter qPCR outcome used for the main analysis by a median of 772 days (range 488–965; *n* = 200). Maternal qPCR was performed in 126 dams. Relative to daughter birth, maternal qPCR occurred before birth in 112 dams, on the day of birth in one dam, and after birth in 13 dams; the median timing was 213.5 days before birth (range 422 days before to 212 days after birth). The median interval from maternal qPCR to the daughter qPCR outcome used for the main analysis was 714 days (range 303–938). These sampling intervals were included as covariates in the maternal models where applicable. Maternal BLV test status in relation to daughter BLV qPCR outcome is summarized in Table 1.

### 2.2. Association Between Maternal BLV Infection Markers and Daughter qPCR Detection

Among daughters with a qPCR outcome available for the main analysis, BLV proviral DNA was detected in 26/123 (21.1%) daughters of ELISA-positive dams and 7/77 (9.1%) daughters of ELISA-negative dams. In an age- and farm-adjusted model, maternal ELISA positivity was associated with higher estimated odds of daughter BLV proviral DNA detection (OR 2.74, 95% CI 1.10–6.83; *p* = 0.031). In the joint model additionally including daughter *BoLA-DRB3* non-redundant epitope breadth and the maternal ELISA-to-daughter sampling interval, the estimate was similar but less precise (OR 2.80, 95% CI 0.98–8.03; *p* = 0.056; *n* = 154; 27 events; Table 2).

Maternal PVL analyses were restricted to dams that actually underwent qPCR. Overall, BLV proviral DNA was detected in 112/126 qPCR-tested dams and was not detected in 14/126. Daughter BLV proviral DNA detection occurred in 24/112 (21.4%) daughters of dams with detectable provirus and 2/14 (14.3%) daughters of dams in which BLV proviral DNA was not detected (unadjusted Fisher *p* = 0.733). In the joint complete-case model, binary maternal qPCR status was not associated with daughter qPCR detection (OR 0.95, 95% CI 0.13–6.75; *p* = 0.961; *n* = 95; Table 2).

Among the 126 qPCR-tested dams, maternal PVL ranged from 0 to 3.234 (median 0.64). Median maternal PVL was 1.187 among daughters who were qPCR-detected and 0.595 among daughters who were qPCR-undetected. Because maternal qPCR testing was strongly dependent on maternal ELISA status, the joint PVL model was restricted to the uniformly qPCR-tested ELISA-positive subset with complete daughter epitope-breadth data (*n* = 95; 21 daughter qPCR-detected events). Maternal PVL showed a positive but imprecise association with daughter qPCR detection (OR 1.94 per 1.0-unit PVL increase, 95% CI 0.89–4.22; *p* = 0.096). A sensitivity model including all qPCR-tested dams and adjusted for age and farm gave a similar direction of association (OR 2.23, 95% CI 1.15–4.34; *p* = 0.018), but this estimate remains susceptible to the non-random maternal testing pattern. An additional sensitivity analysis restricted maternal PVL measurements to tests obtained on or before daughter birth; in that joint complete-case subset (*n* = 82; 21 daughter qPCR-detected events), the maternal PVL estimate was OR 1.82 (95% CI 0.82–4.04; *p* = 0.139). Farm-stratified results and additional age- and farm-adjusted maternal-marker analyses are provided in Supplementary Table S4.

### 2.3. BoLA-DRB3 Genotyping and Allele Distribution in Daughters

*BoLA-DRB3* genotyping was successful in 199 daughters; two samples failed genotyping, yielding 398 allele assignments. Fifty-three distinct allele labels were identified. The allele-frequency distribution was strongly skewed: the four most frequent alleles, BoLA-DRB3*001:01 (138/398; 34.7%), BoLA-DRB3*014:01:01 (66/398; 16.6%), BoLA-DRB3*010:01 (35/398; 8.8%), and BoLA-DRB3*011:01 (29/398; 7.3%), accounted for 268/398 assignments (67.3%), whereas 43/53 allele labels (81.1%) occurred three or fewer times. Because the study was based on a convenience sample from five herds and was not designed as a population-genetic survey, no specific evolutionary or demographic mechanism was inferred for this distribution.

### 2.4. BoLA-DRB3 Epitope Predictions and Genotype-Level Breadth

Published BLV Gag epitope-prediction data [30] were available for 348 of 398 allele assignments representing 18 allele labels, whereas prediction data were unavailable for 50 assignments representing 35 allele labels (Table 3). At the animal level, prediction information was available for both alleles in 155 daughters, for exactly one allele in 38 daughters, and for neither allele in 6 daughters. Genotype-level epitope breadth was calculated only when prediction information was available for both alleles. Missing prediction information was treated as missing. A detailed summary of *BoLA-DRB3* prediction completeness and analysis-specific sample sizes is provided in Supplementary Table S6.

Genotype-level epitope breadth was defined as the number of unique predicted BLV Gag epitopes in the non-redundant union across both alleles. Among 155 daughters with complete prediction information, breadth ranged from 3 to 11 unique epitopes (median, 7). At least one predicted epitope was shared by both alleles in 143/155 genotypes (92.3%).

### 2.5. Association of BoLA-DRB3 Epitope Breadth with Daughter BLV Outcomes

Among 154 daughters with complete epitope-prediction information and a qPCR outcome available for the main analysis, BLV proviral DNA was detected in 27. Greater non-redundant epitope breadth was nominally associated with lower odds of BLV proviral DNA detection after adjustment for age and farm (OR 0.76 per additional unique epitope, 95% CI 0.60–0.97; *p* = 0.025; BH-FDR q = 0.162; Figure 1). For daughter ELISA, 32/154 daughters with complete prediction information were seropositive for BLV; greater breadth was also nominally associated with lower odds of BLV seropositivity after adjustment for assay-specific age and farm (OR 0.80, 95% CI 0.65–0.98; *p* = 0.033; q = 0.162). Neither association met q < 0.05 after correction across the defined 12-test *BoLA* FDR family (Supplementary Table S5).

To distinguish factors associated with BLV detection from those associated with viral burden among detected animals, PVL was analyzed separately among daughters with detectable BLV proviral DNA. In 27 BLV-positive daughters with complete epitope-breadth information, the age- and farm-adjusted multiplicative PVL ratio per additional unique epitope was 1.26 (95% CI 0.88–1.82; *p* = 0.211; q = 0.316). Predicted epitope breadth was not associated with PVL magnitude. This conditional result is consistent with the lack of a significant overall epitope-number/PVL relationship previously reported among BLV-infected animals [30].

### 2.6. Sensitivity Analyses of BoLA-DRB3*001:01, BoLA-DRB3*014:01:01, and Predicted Coverage of Epitopes 1A and 2

BoLA-DRB3*001:01 carrier status was analyzed as a genotype-based sensitivity feature that did not require complete epitope-prediction information. Among daughters with available qPCR results and successful genotyping, BLV proviral DNA was detected in 21/92 carriers (22.8%) and 12/106 non-carriers (11.3%); the unadjusted Fisher exact *p* value was 0.036. After adjustment for age and farm, the estimate was attenuated and imprecise (OR 1.99, 95% CI 0.89–4.44; *p* = 0.092; q = 0.220; *n* = 198). No associations of BoLA-DRB3*001:01 with BLV seropositivity or conditional PVL met the BH-FDR threshold.

BoLA-DRB3*014:01:01 was the second most frequent allele in the cohort (66/398 allele assignments; 16.6%) and has previously been associated with lower PVL or resistance-associated BLV phenotypes [29,31]. Among daughters with available qPCR results and successful genotyping, BLV proviral DNA was detected in 3/46 BoLA-DRB3*014:01:01 carriers (6.5%) and 30/152 non-carriers (19.7%; unadjusted Fisher exact *p* = 0.041). After adjustment for age and farm, carriers had lower estimated odds of BLV qPCR detection (OR = 0.29, 95% CI 0.08–1.02; *p* = 0.053; q = 0.162). BLV seropositivity was observed in 5/46 carriers (10.9%) and 38/152 non-carriers (25.0%); the adjusted estimate was OR = 0.37 (95% CI 0.13–1.02; *p* = 0.054; q = 0.162). Among daughters with detectable proviral DNA, only three BoLA-DRB3*014:01:01 carriers were available; their PVL values were 0.002, 0.072, and 0.084 (median 0.072), and the adjusted conditional PVL estimate was highly imprecise (PVL ratio = 0.21, 95% CI 0.006–7.62; *p* = 0.391; q = 0.469). Thus, the direction of the qPCR and ELISA estimates was consistent with a resistance-associated phenotype, but no BoLA-DRB3*014:01:01 association met the BH-FDR threshold in this cohort.

Predicted genotype coverage of both epitopes 1A and 2 was not associated with PVL detection (age- and farm-adjusted OR 0.44, 95% CI 0.15–1.29; *p* = 0.135; q = 0.237) or ELISA seropositivity (OR 0.78, 95% CI 0.29–2.11; *p* = 0.627; q = 0.684). Among daughters with detectable BLV proviral DNA, predicted coverage of both epitopes was not associated with PVL magnitude (multiplicative PVL ratio 0.36, 95% CI 0.05–2.38; *p* = 0.288; q = 0.383). The complete set of *BoLA-DRB3* sensitivity analyses is summarized in Table 4.

## 3. Discussion

This study integrates longitudinal BLV testing, maternal infection markers, and daughter *BoLA-DRB3* variation to characterize epidemiologic and host factors associated with BLV outcomes during the youngstock period. Three main findings emerged. First, daughters of ELISA-positive dams had higher estimated odds of later BLV proviral DNA detection, while maternal PVL estimates were consistently positive but less precise. Second, most first detections occurred at prebreeding or post-breeding stages rather than at the preweaning stage, indicating that detectable BLV status changes across the rearing period. Third, greater non-redundant predicted *BoLA-DRB3* epitope breadth was nominally associated with lower odds of BLV proviral DNA detection and BLV seropositivity, although these associations did not remain significant after FDR correction and breadth was not associated with PVL magnitude among daughters with detectable BLV provirus. Together, these findings indicate that maternal infection context, exposure during rearing, herd-level factors, and host immunogenetic variation may contribute to heterogeneity in BLV outcomes in dairy youngstock.

Maternal BLV infection markers were associated with differences in the probability of later qPCR detection in daughters. In the age- and farm-adjusted model, daughters of ELISA-positive dams had approximately 2.7-fold higher odds of qPCR detection. The effect estimate remained similar after additional adjustment for daughter *BoLA-DRB3* epitope breadth and the maternal-to-daughter sampling interval, although precision was reduced. Maternal PVL was also higher, on average, in dams of qPCR-detected daughters, and the age- and farm-adjusted sensitivity analysis including all qPCR-tested dams showed a positive association. In the ELISA-positive subset, in which all dams underwent qPCR, the corresponding joint estimate was less precise, whereas binary maternal qPCR status was not associated with daughter qPCR detection. These findings suggest that maternal ELISA status, and potentially maternal PVL, may help identify daughter groups with different likelihoods of later BLV proviral DNA detection. This interpretation is biologically plausible because studies of naturally exposed calves in Japan have documented perinatal BLV infection and identified maternal viral burden as an important risk factor, while high maternal viral load has also been associated with intrauterine infection [14,17]. At the same time, ELISA and PVL represent different dimensions of maternal infection: ELISA provides serologic evidence of infection, whereas PVL quantifies proviral burden at the time of molecular testing [18,19,20]. This distinction, together with the selective maternal qPCR testing pattern in the present cohort, may partly explain why the ELISA association was more stable than the binary qPCR estimate. Because transmission route was not established, however, maternal measures are best interpreted as epidemiologic risk markers rather than evidence of direct maternal transmission. This is consistent with the broader BLV literature, which recognizes multiple concurrent transmission routes in endemic herds, including blood exposure, in utero transmission, colostrum, and milk [32].

The longitudinal component provides important context for that interpretation. Only two daughters had detectable BLV proviral DNA at the preweaning sampling point, whereas 24 were first detected at prebreeding and 12 at post-breeding. Moreover, 34 of the 36 daughters first detected later had at least one earlier qPCR result in which BLV proviral DNA was not detected. Comparable natural-history observations have been reported in Argentinean dairy cattle, where BLV infection status evolved from birth through the period preceding first lactation [25]. Experimental inoculation studies have likewise shown that molecular and serologic detection do not occur simultaneously and that substantial between-animal variation exists in the establishment and magnitude of PVL [33]. The temporal pattern observed in the present cohort is compatible with cumulative exposure during rearing, but it is also compatible with earlier infection that remained below the assay detection threshold. The possibility of limited detectability at low proviral copy numbers is supported by an inter-laboratory comparison of 11 molecular assays for BLV proviral DNA, including the multiplex qPCR used in the present study, in which diagnostic sensitivity ranged from 74% to 100% and inter-assay agreement depended on the number of BLV copies present in the sample [19]. Samples with low proviral copy numbers showed the greatest variability in detection, with some assays failing to detect BLV DNA that was identified by other methods [19]. Thus, an earlier negative molecular result should be interpreted in the context of both infection dynamics and analytical sensitivity. Horizontal exposure later in the rearing period is also biologically plausible because BLV transmission depends on the transfer of infected lymphocytes and has been linked to blood-contaminated procedures and other management-related exposures [5,6,7,8,9,10,32]. Together, these observations reinforce the value of repeated testing for characterizing BLV infection dynamics in youngstock.

The *BoLA-DRB3* findings provide complementary evidence that host immunogenetic variation may contribute to heterogeneity in BLV outcomes. Genotype-level epitope breadth was associated with lower odds of BLV proviral DNA detection and ELISA positivity for anti-BLV antibodies before correction for multiple testing. In contrast, epitope breadth was not associated with lower PVL among qPCR-detected daughters, suggesting that determinants of detectable infection and determinants of viral burden after infection may not be identical. Associations between *BoLA-DRB3* variation and several BLV phenotypes have been reported across different stages of infection. In Argentinean Holsteins, DRB3 alleles were associated with low- and high-PVL infection profiles [34], and experimental infection of calves showed divergence in PVL kinetics after seroconversion according to resistance- and susceptibility-associated DRB3 backgrounds [16]. Population studies in Japan, Egypt, and China have likewise demonstrated associations between specific *BoLA-DRB3* alleles and BLV infection or PVL [26,29,31]. Importantly, the spectrum of *BoLA-DRB3* alleles associated with BLV infection and PVL differs among cattle populations, highlighting the potential influence of population genetic background and epidemiological context [26,29,31]. Such heterogeneity argues against treating a single allele as universally protective or susceptible and supports evaluation of host genetics in the context of population structure and viral and environmental background. The present BoLA-DRB3*014:01:01 result is notable in this context. This allele was the second most frequent in the cohort, and carriers had lower observed frequencies of qPCR detection and BLV seropositivity than non-carriers. The age- and farm-adjusted estimates were in the same direction, although confidence intervals included the null and the associations did not remain significant after FDR correction. The direction of these estimates is concordant with resistance-associated findings for BoLA-DRB3*014:01:01 in Japanese and Chinese Holstein populations [29,31]. Among daughters with detectable BLV proviral DNA, however, only three *014:01:01 carriers were available, precluding a precise estimate of its association with PVL magnitude. The genotype-level epitope-breadth analysis provides a prediction-based perspective complementary to these allele-specific observations. A broader predicted peptide-binding repertoire could plausibly expand the range of BLV-derived peptides available for CD4+ T-cell recognition; however, in silico binding predictions do not establish actual peptide presentation or T-cell responses [30]. Accordingly, the epitope-breadth findings should be viewed as hypothesis-generating evidence that links *BoLA* diversity to antigen-presentation potential, rather than as proof of a protective mechanism. Together, these results support continued investigation of both allele-specific and functional *BoLA-DRB3* effects in larger, genetically diverse longitudinal cohorts.

From a herd-management perspective, the findings are consistent with a layered rather than single-marker approach. Maternal ELISA status may help identify daughter groups for closer follow-up, while longitudinal qPCR testing captures changes in detectable infection status over time. PVL can provide additional information for relative transmission-risk prioritization, particularly in high-prevalence herds where interventions may need to be staged. This interpretation is consistent with field studies in which preferential management or removal of high-PVL cattle was associated with reductions in herd prevalence or incidence [22,35], as well as with applied control programs combining serology, lymphocyte measures, PVL, and management interventions [24]. A broader review of BLV control concluded that high-PVL cattle are generally more likely to transmit infection, but also emphasized that successful eradication has relied on combinations of management, segregation, and culling rather than on a single diagnostic marker [32]. Although cattle with very low PVL have shown low transmission risk under some experimental and field conditions [23], low transmission risk at a given time point should not be equated with permanent epidemiologic irrelevance. These findings suggest that maternal markers and PVL may contribute to risk stratification within a broader BLV control framework rather than serving as stand-alone criteria for exclusion, retention, or eradication decisions.

Several limitations should be considered when interpreting these results. The study included five convenience-sampled herds, and unmeasured management practices may differ among farms despite adjustment with farm fixed effects. Maternal qPCR testing was strongly dependent on maternal ELISA status, limiting direct inference from maternal PVL comparisons; dams were also not genotyped, leaving residual shared-genetic confounding possible. The number of BLV qPCR-detected daughters was modest, leading to wide confidence intervals in some multivariable models. No systematic pre-colostrum daughter testing or dam-daughter viral sequence concordance was available, so transmission route cannot be assigned. For the *BoLA* analysis, published epitope predictions were unavailable for 50 of 398 allele assignments and were based on in silico predictions that require experimental validation.

Taken together, the findings show that BLV outcomes during the youngstock period reflect a combination of maternal infection context, cumulative exposure during rearing, and host immunogenetic variation. Maternal BLV markers may help identify young animals with a higher probability of later BLV detection, whereas the *BoLA-DRB3* findings highlight potentially relevant host-genetic heterogeneity that requires validation in larger cohorts. The international literature also indicates that the magnitude and direction of individual *BoLA-DRB3* associations can vary among cattle populations [26,29,31,34], making external validation across distinct genetic and management backgrounds particularly important. Longitudinal studies integrating maternal and daughter genotypes, repeated molecular testing, viral sequence analysis, and detailed herd-management data will be important for separating transmission pathways from inherited susceptibility and for translating these associations into effective BLV control strategies.

## 4. Materials and Methods

### 4.1. Dam-Daughter Cohort and Maternal BLV Testing

The source cohort comprised 254 daughter animals enrolled across five dairy farms. The analytical cohort included 201 dam–daughter pairs for which the required maternal and daughter records and archived biological material were available. Animals not included in the linked analytic cohort primarily lacked archived material required for downstream DNA-based analyses or were no longer available because of routine removal or culling during follow-up. Farms and animals were enrolled by convenience rather than probability sampling.

Maternal BLV screening was conducted through routine CentralStar Cooperative Dairy Herd Information services. Milk samples were analyzed by BLV ELISA. All 123 ELISA-positive dams underwent blood qPCR testing for BLV proviral load (PVL); whereas only 3 of 78 ELISA-negative dams underwent qPCR. Consequently, maternal qPCR was available for 126/201 dams, and the remaining 75 dams were classified as not tested. The dates of maternal ELISA and qPCR testing were retained to quantify their temporal relationship to daughter birth and daughter sampling.

### 4.2. Daughter Sampling and Definition of the qPCR Outcome

Whole-blood samples were collected from daughters by coccygeal or jugular venipuncture into EDTA tubes, using a new sterile hypodermic needle for each animal. Samples were transported on ice to the diagnostic laboratory. Daughters were scheduled for longitudinal blood collection at three developmental stages: preweaning, prebreeding, and post-breeding; preweaning samples were collected after colostrum intake. For the main analysis, daughter BLV qPCR status was defined using the latest available later-youngstock sample. Post-breeding results were used when available; for six daughters that were culled before post-breeding sampling, the most recent prebreeding result was used instead. Preweaning results were not used to define this outcome. This provided qPCR outcomes for 200 daughters (194 post-breeding and 6 prebreeding); one daughter had no later-youngstock qPCR result. Daughter age at each assay was calculated from the date of birth and sample-collection date.

### 4.3. Serological and Molecular Diagnosis of BLV Infection

Anti-BLV antibodies were detected using the IDEXX Leukosis Milk Screening Ab Test (catalog no. P02210-10; IDEXX Laboratories, Westbrook, ME, USA), applied by AntelBio/CentralStar using a long-standing off-label protocol adapted for individual-animal milk and blood-derived samples. For milk samples, optical density (OD) values < 0.10 were classified as negative, 0.10 to <0.30 as suspect, and ≥0.30 as positive. For blood/plasma samples, OD values < 0.50 were classified as negative, 0.50 to <1.00 as suspect, and ≥1.00 as positive. For statistical analyses, suspect results were grouped with negative results [21,33,35].

For BLV molecular testing, genomic DNA was extracted from whole-blood samples using the MagMAX CORE Nucleic Acid Purification Kit (Life Technologies Corporation, Austin, TX, USA) according to the manufacturer’s instructions; no separate DNA concentration measurement was performed prior to the diagnostic qPCR. BLV proviral DNA was quantified using the commercially available AntelBio STRATA-G BLV PCR assay (CentralStar Cooperative, Inc., Lansing, MI, USA) in a multiplex quantitative real-time PCR format. The assay simultaneously targets a highly conserved region of the BLV *pol* gene with a FAM-labeled TaqMan probe and bovine *beta-actin* with a Cy5-labeled probe, and includes an exogenous internal amplification control to monitor PCR inhibition. Reactions were performed in a total volume of 25 µL using 4 µL of extracted DNA on an Applied Biosystems 7500 Fast Real-Time PCR System (Applied Biosystems, Foster City, CA, USA). Cycling comprised an initial denaturation at 95 °C followed by 40 cycles of 95 °C for 15 s and 60 °C for 60 s. Positive and negative plasmid controls were included in each run. BLV and *beta-actin* copy numbers were calculated from precision DNA standards quantified by droplet digital PCR and prepared as 10-fold serial dilutions extending to one copy. BLV proviral DNA was considered detected when the amplification signal exceeded the 3% manual threshold established within the assay calibration [15,21]. PVL was calculated as the ratio of BLV *pol* copy number to bovine *beta-actin* copy number measured in the same multiplex reaction (PVL = BLV copies/bovine *beta-actin* copies) and was analyzed as a continuous quantitative value. For descriptive laboratory reporting, PVL values were categorized as undetected (0.000), low (>0.000–0.499), moderate (0.500–0.999), and high (≥1.000). These categories were not interpreted as universal biological thresholds of transmission risk [36,37,38].

### 4.4. BoLA-DRB3 Genotyping

*BoLA-DRB3* genotyping was attempted in 201 daughters. A 297 bp fragment of exon 2 was amplified using primers HL030 (5′-AGATCTATCCTCTCTCTGCAGCACATTTCC-3′) and HL031 (5′-CGCGCTCACCTCGCCGCT-3′) [39]. PCR reactions were performed in 30 µL containing 1× Pol Buffer A, 2.5 mM MgCl2, 115 µM of each dNTP, 0.3 µM of each primer, 0.4 U OptiTaq DNA Polymerase (EURx, Gdansk, Poland), and approximately 12 ng genomic DNA. Cycling consisted of 94 °C for 3 min; 35 cycles of 94 °C for 20 s, 60 °C for 20 s, and 72 °C for 1 min; and a final extension at 72 °C for 5 min. PCR products were purified with ExoSAP-IT (Thermo Fisher Scientific, Waltham, MA, USA). Each amplicon was subjected to Sanger sequencing using HL030 in the forward direction and HL031 and HL032 (5′-TCGCCGCTGCACAGTGAAACTCTC-3′) in the reverse direction, generating one forward and two reverse sequence reads per amplicon [39,40].

### 4.5. Sequence-Based Typing of BoLA-DRB3 Alleles

Forward and reverse Sanger reads were trimmed to remove primer-derived sequence and aligned in Geneious Prime (ver. 2026.0.2; Biomatters Ltd., Auckland, New Zealand). Allele assignment used the resulting 249 bp informative exon 2 sequence; heterozygous positions were represented with IUPAC ambiguity codes. Consensus sequences were compared with an archived IPD-MHC *BoLA-DRB3* reference set using the Haplofinder workflow and were cross-checked against a custom Geneious Prime (ver. 2026.0.2; Biomatters Ltd., Auckland, New Zealand) BLAST database with Megablast for highly similar sequences (low-complexity filter; scoring 1/-2; maximum E-value threshold < 0.05) [40]. The IPD-MHC reference set comprised 389 *BoLA-DRB3* sequences and corresponded to IPD-MHC release 3.11.0.0 (July 2023), downloaded in July 2023, consistent with the reference set used in our previous epitope-prediction study [30].

### 4.6. Prediction of BLV Gag Epitope Binding and Genotype-Level Breadth

*BoLA-DRB3*-restricted BLV Gag epitope predictions were obtained from a previous study using NetBoLAIIpan version 1.0 (DTU Health Tech, Technical University of Denmark, Kongens Lyngby, Denmark) [30]. In that framework, percentile rank was calculated relative to 100,000 random natural peptides. For the present analysis, predictions with %Rank ≤ 5.0 were retained as epitope-binding predictions, following the epitope-retention criterion applied in the previous study [30]. Within the retained predictions, %Rank < 1.0 was classified as strong binding and %Rank ≥ 1.0 to <5.0 as weak binding. Prediction information corresponding to the daughter alleles was available for 18 *BoLA-DRB3* allele labels. For genotype-level analyses, prediction information was required for both alleles. Genotype-level epitope breadth was defined as the number of unique predicted BLV Gag epitopes in the non-redundant union of the two allele-specific repertoires. If prediction information was unavailable for either allele, genotype-level epitope breadth was treated as missing. Sensitivity analyses additionally evaluated BoLA-DRB3*001:01 carrier status, BoLA-DRB3*014:01:01 carrier status, and predicted genotype coverage of both epitopes 1A and 2.

### 4.7. Statistical Analysis

Statistical analyses were performed in Python 3.13.5 (Python Software Foundation, Beaverton, OR, USA) using statsmodels 0.14.6, SciPy 1.17.0, and NumPy 2.3.5. Continuous variables were summarized using medians with interquartile ranges and ranges where appropriate; categorical variables were summarized as counts and percentages. Farm was modeled as a fixed categorical effect because only five convenience-sampled herds were included. Daughter age was included in main binary-outcome models, and maternal-to-daughter sampling interval was included in joint maternal models. Age and interval were scaled per 100 days for regression reporting.

The primary maternal ELISA model used logistic regression for daughter BLV qPCR detection and included maternal ELISA status, daughter age, daughter non-redundant epitope breadth, farm fixed effects, and the maternal ELISA-to-daughter qPCR interval. Because maternal qPCR testing was strongly dependent on maternal ELISA status, the joint PVL analysis was restricted to ELISA-positive dams with qPCR results and complete covariate data. Maternal PVL was modeled on its original continuous scale. Farm-stratified sensitivity analyses were used to assess robustness to herd structure. The logistic model was specified as logit[P(Y = 1)] = β0 + β1(maternal marker) + β2(daughter age/100 days) + β3(epitope breadth) + β4(sampling interval/100 days) + farm fixed-effect terms. Here, Y = 1 denotes detection of BLV proviral DNA in the daughter qPCR outcome used for the main analysis, and Y = 0 denotes no detection of BLV proviral DNA. P(Y = 1) therefore represents the probability of daughter BLV proviral DNA detection. β0 is the intercept, and β1-β4 are the regression coefficients for the maternal BLV marker, daughter age, daughter epitope breadth, and maternal-to-daughter sampling interval, respectively. Daughter age and sampling interval were divided by 100 so that their ORs represent a 100-day increase, whereas epitope breadth was modeled per one additional unique predicted BLV Gag epitope. The maternal marker term represented maternal ELISA status in the ELISA model, continuous maternal PVL in the PVL model, and detected versus undetected maternal qPCR status in the binary qPCR model. Farm 1 served as the reference category. An additional maternal PVL sensitivity analysis restricted maternal qPCR measurements to those obtained on or before daughter birth.

The *BoLA* multiple-testing family comprised four genetic features (BoLA-DRB3*001:01 carrier status, BoLA-DRB3*014:01:01 carrier status, non-redundant genotype-level epitope breadth, and predicted coverage of both epitopes 1A and 2) evaluated across three outcomes (daughter qPCR detection, daughter BLV seropositivity, and PVL conditional on qPCR detection). Binary qPCR and ELISA outcomes were analyzed by logistic regression adjusted for assay-specific daughter age and farm fixed effects. PVL magnitude was analyzed only among daughters with detectable BLV provirus using linear regression of log10(PVL), adjusted for age and farm, with HC3 heteroskedasticity-robust standard errors; exponentiation on base 10 yielded multiplicative PVL ratios. Benjamini–Hochberg FDR correction was applied across the resulting 12 association tests. Fisher exact tests were used only for selected unadjusted sparse 2 × 2 descriptive comparisons.

Model diagnostics were assessed for the multivariable maternal models. Maternal BLV qPCR status and PVL were analyzed in separate models, with farm included as a fixed categorical effect. Model-fit summaries are provided in Supplementary Table S7. The analytic cohort included 195 unique dams; six dams contributed two daughters.

## 5. Conclusions

Maternal BLV infection markers were associated with later BLV outcomes in dairy youngstock, although these associations should not be interpreted as evidence of direct maternal transmission. Longitudinal qPCR testing showed that BLV proviral DNA was frequently first detected later in the rearing period, emphasizing the value of repeated monitoring rather than reliance on a single test result. Daughter *BoLA-DRB3* variation was also associated with heterogeneity in BLV outcomes, although the retained *BoLA* associations did not remain significant after correction for multiple testing. Together, these findings indicate that BLV outcomes in youngstock are associated with maternal infection context, exposure during rearing, and host immunogenetic variation. Repeated diagnostic testing, herd-level biosecurity, and further characterization of host genetic susceptibility may contribute to improved BLV surveillance and control strategies.

## Figures and Tables

**Figure 1:**
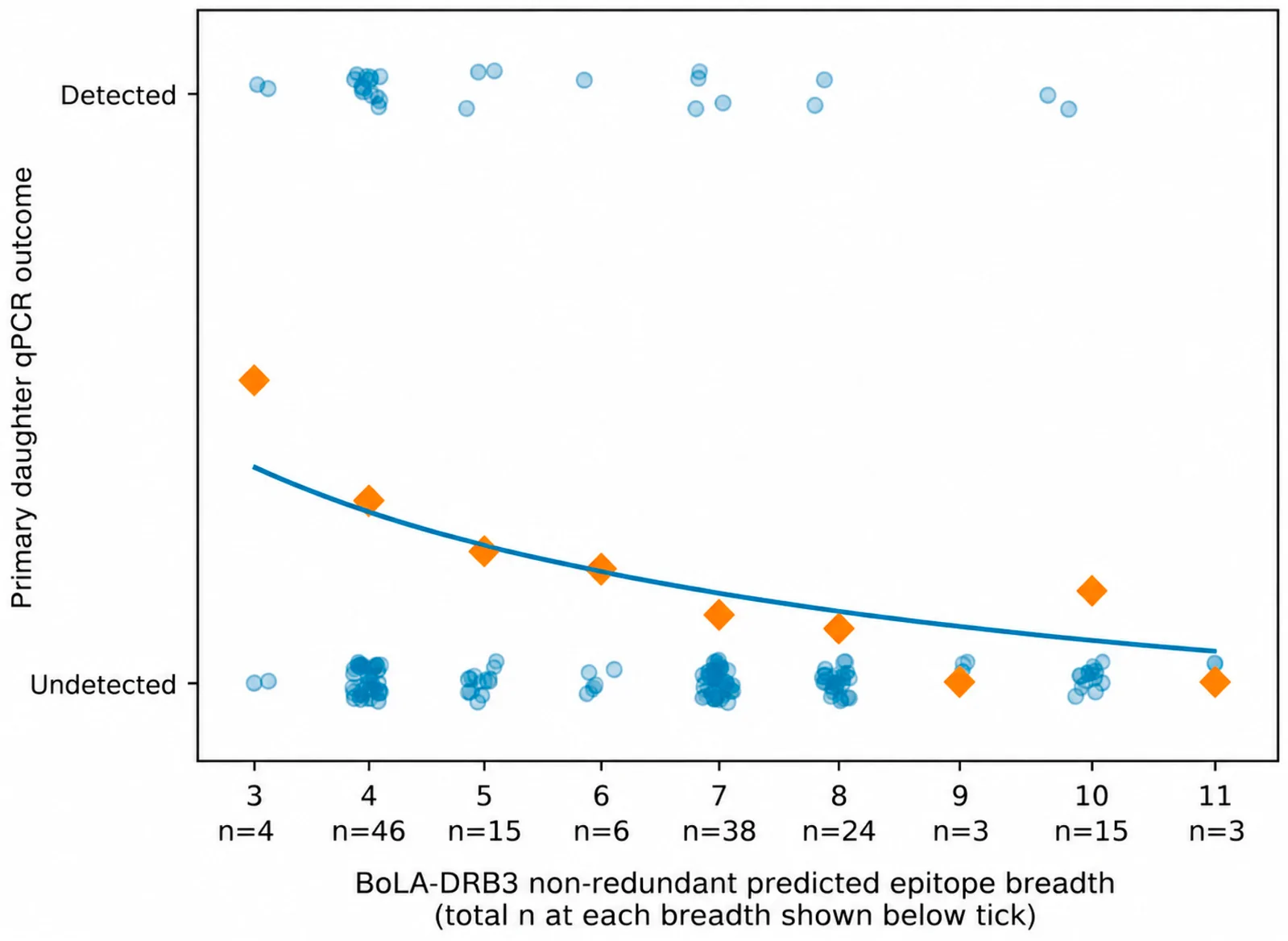
Association between daughter *BoLA-DRB3* non-redundant predicted epitope breadth and BLV proviral DNA detection by qPCR. Blue circles represent individual daughter qPCR outcomes shown with jitter and transparency. Orange diamonds indicate the observed proportion of daughters with detectable BLV proviral DNA at each epitope-breadth value, and the blue solid curve shows the marginal age- and farm-adjusted fitted probability from logistic regression. The adjusted OR was 0.76 per additional unique predicted epitope (95% CI 0.60–0.97; nominal *p* = 0.025; BH-FDR *q* = 0.162; *n* = 154, including 27 daughters with detectable BLV proviral DNA). OR, odds ratio; CI, confidence interval; *p*, nominal *p* value; *q*, Benjamini–Hochberg FDR-adjusted *p* value; BH-FDR, Benjamini–Hochberg false discovery rate; *n*, number of daughters.

**Table 1:** Maternal BLV test status and daughter BLV qPCR outcome used for the main analysis. Maternal qPCR categories distinguish actual testing from tests not performed. Percentages are calculated among daughters with an available qPCR outcome within each maternal-status row; missing outcomes are shown separately.

Maternal Marker	Dam Status	Daughter BLV Proviral DNA Detected	Daughter BLV Proviral DNA Not Detected	Daughter qPCR Missing	Total Dams
ELISA	Positive	26 (21.1%)	97 (78.9%)	0	123
ELISA	Negative	7 (9.1%)	70 (90.9%)	1	78
qPCR	Detected	24 (21.4%)	88 (78.6%)	0	112
qPCR	Undetected	2 (14.3%)	12 (85.7%)	0	14
qPCR	Not performed	7	67	1	75

Maternal qPCR was performed in 126/201 dams (62.7%). All 123 ELISA-positive dams were qPCR-tested; only 3/78 ELISA-negative dams were qPCR-tested. Dams without a qPCR result were classified as not tested and were excluded from analyses requiring maternal qPCR or PVL. Cross-tabulation of maternal ELISA and qPCR testing status is provided in Supplementary Table S3. Abbreviations: BLV, bovine leukemia virus; ELISA, enzyme-linked immunosorbent assay; qPCR, quantitative polymerase chain reaction.

**Table 2:** Multivariable logistic regression models of maternal BLV infection markers and daughter BLV proviral DNA detection by qPCR.

Predictor	Beta	SE	Wald z	*p*	OR	95% CI
Model A: maternal ELISA joint model (*n* = 154; 27 events)						
Maternal ELISA positive vs. negative	1.030	0.538	1.915	0.056	2.80	0.98–8.03
Daughter age, per 100 days	0.948	0.667	1.421	0.155	2.58	0.70–9.54
Daughter epitope breadth, per epitope	−0.287	0.127	−2.265	0.024	0.75	0.58–0.96
Maternal ELISA-to-daughter interval, per 100 days	−0.295	0.253	−1.164	0.244	0.74	0.45–1.22
Farm 2 vs. Farm 1	0.224	0.677	0.331	0.741	1.25	0.33–4.72
Farm 3 vs. Farm 1	−0.031	1.123	−0.028	0.978	0.97	0.11–8.76
Farm 4 vs. Farm 1	0.242	0.747	0.324	0.746	1.27	0.29–5.51
Farm 5 vs. Farm 1	−1.563	0.978	−1.599	0.110	0.21	0.03–1.42
Model B: maternal PVL; qPCR-verified ELISA-positive dams (*n* = 95; 21 events)						
Maternal PVL, per 1.0-unit increase	0.661	0.397	1.667	0.096	1.94	0.89–4.22
Daughter age, per 100 days	0.389	0.805	0.484	0.628	1.48	0.30–7.15
Daughter epitope breadth, per epitope	−0.178	0.149	−1.193	0.233	0.84	0.62–1.12
Maternal qPCR-to-daughter interval, per 100 days	−0.086	0.338	−0.254	0.800	0.92	0.47–1.78
Farm 2 vs. Farm 1	0.268	0.809	0.331	0.741	1.31	0.27–6.39
Farm 3 vs. Farm 1	0.721	1.327	0.543	0.587	2.06	0.15–27.69
Farm 4 vs. Farm 1	0.240	0.898	0.268	0.789	1.27	0.22–7.40
Farm 5 vs. Farm 1	−1.361	1.383	−0.984	0.325	0.26	0.02–3.86
Model C: binary maternal qPCR sensitivity model (*n* = 95; 21 events)						
Maternal qPCR detected vs. undetected	−0.048	0.999	−0.048	0.962	0.95	0.13–6.75
Daughter age, per 100 days	0.625	0.764	0.818	0.413	1.87	0.42–8.36
Daughter epitope breadth, per epitope	−0.231	0.148	−1.564	0.118	0.79	0.59–1.06
Maternal qPCR-to-daughter interval, per 100 days	−0.240	0.328	−0.732	0.464	0.79	0.41–1.49
Farm 2 vs. Farm 1	0.251	0.785	0.320	0.749	1.29	0.28–5.98
Farm 3 vs. Farm 1	0.288	1.388	0.207	0.836	1.33	0.09–20.26
Farm 4 vs. Farm 1	0.046	0.887	0.052	0.959	1.05	0.18–5.95
Farm 5 vs. Farm 1	−1.888	1.426	−1.324	0.186	0.15	0.01–2.48

Outcome: later-youngstock daughter qPCR result used for the main analysis; post-breeding was prioritized over prebreeding. Farm was modeled with fixed effects. Models B and C were restricted to ELISA-positive dams with an actual maternal qPCR result and complete daughter epitope-breadth data. Missing epitope-prediction information was treated as missing. Abbreviations and symbols: beta, regression coefficient; SE, standard error; *z*, Wald z statistic; *p*, two-sided *p* value; OR, odds ratio; CI, confidence interval; *n*, number of daughters included in the model; PVL, proviral load.

**Table 3:** Frequency of *BoLA-DRB3* allele assignments and availability of published BLV epitope-prediction information.

*BoLA-DRB3* Allele	*n*	Frequency (%)	Prediction Data	No. of Predicted Epitopes	Predicted BLV Epitopes
*001:01	138	34.7	Yes	4	2, 6, 1A, 4A
*014:01:01	66	16.6	Yes	7	2, 10, 1A, 1B, 4A, 4C, 11B
*010:01	35	8.8	Yes	8	3, 6, 7, 8, 14, 4A, 12A, 13B
*011:01	29	7.3	Yes	3	1B, 4C, 11B
*015:01	22	5.5	Yes	5	3, 5, 6, 4A, 13A
*031:01	15	3.8	Yes	5	2, 6, 1A, 1B, 4A
*027:03	9	2.3	Yes	6	2, 7, 1A, 1B, 4B, 4C
*044:01	9	2.3	Yes	6	3, 7, 8, 4A, 4B, 12A
*012:01	5	1.3	Yes	5	3, 7, 8, 4B, 4C
*009:02	4	1.0	Yes	7	2, 5, 10, 1A, 1B, 4A, 4B
*011:04	3	0.8	No		—
*011:1	3	0.8	No		—
*016:01	3	0.8	Yes	5	3, 7, 8, 4B, 11A
*024:25	3	0.8	No		—
*081:01	3	0.8	Yes	6	2, 3, 6, 1A, 4A, 12A
*117:01	3	0.8	No		—
*118:01	3	0.8	No		—
*002:01	2	0.5	Yes	9	2, 3, 5, 6, 7, 1A, 1B, 4A, 12A
*007:01	2	0.5	Yes	7	2, 6, 9, 1A, 4A, 4C, 11B
*010:04	2	0.5	Yes	5	3, 6, 14, 4A, 12A
*012:04	2	0.5	No		—
*013:04	2	0.5	No		—
*027:10	2	0.5	No		—
*075:03	2	0.5	Yes	7	2, 5, 1A, 1B, 4A, 4C, 13A
*109:01	2	0.5	No		—
*130:01	2	0.5	No		—
*007:04	1	0.3	No		—
*011:05	1	0.3	No		—
*020:01:01	1	0.3	Yes	3	7, 4C, 12B
*022:11	1	0.3	No		—
*024:33	1	0.3	No		—
*027:09	1	0.3	No		—
*027:14	1	0.3	No		—
*028:03	1	0.3	No		—
*031:00	1	0.3	No		—
*031:04	1	0.3	No		—
*036:00	1	0.3	No		—
*045:01	1	0.3	No		—
*064:01	1	0.3	No		—
*064:02	1	0.3	No		—
*064:03	1	0.3	No		—
*068:01	1	0.3	No		—
*089:01	1	0.3	Yes	5	2, 3, 6, 1A, 4A
*096:01	1	0.3	No		—
*100:01	1	0.3	No		—
*112:02	1	0.3	No		—
*112:03	1	0.3	No		—
*116:01	1	0.3	No		—
*116:02	1	0.3	No		—
*119:01	1	0.3	No		—
*138:01	1	0.3	No		—
*161:01	1	0.3	No		—
*162:01	1	0.3	No		—

Genotyping was successful in 199 daughters, yielding 398 assignments. Published epitope-prediction information was available for 348 assignments (18 allele labels) and unavailable for 50 assignments (35 labels). Alleles without prediction information were retained for allele-frequency summaries but treated as missing in genotype-level epitope-breadth analyses. In this table, the locus name is specified in the header, so entries are abbreviated as *001:01, *014:01:01, etc. The asterisk (*) is part of the BoLA-DRB3 allele nomenclature and does not indicate a footnote or statistical significance. n, number of allele assignments; Epitope n, number of predicted BLV epitopes; —, published epitope-prediction data not available.

**Table 4:** *BoLA-DRB3* sensitivity analyses with effect sizes and Benjamini–Hochberg FDR correction.

*BoLA* Feature	Outcome	*n*	Effect Estimate	95% CI	*p*	*q*
*001:01 carrier	qPCR detection	198	OR 1.99	0.89–4.44	0.092	0.220
*001:01 carrier	BLV seropositivity	198	OR 1.70	0.84–3.44	0.138	0.237
*001:01 carrier	PVL	33	PVL ratio 1.08	0.20–5.80	0.933	0.933
*014:01:01 carrier	qPCR detection	198	OR 0.29	0.08–1.02	0.053	0.162
*014:01:01 carrier	BLV seropositivity	198	OR 0.37	0.13–1.02	0.054	0.162
*014:01:01 carrier	PVL	33	PVL ratio 0.21	0.006–7.62	0.391	0.469
Epitope breadth	qPCR detection	154	OR 0.76	0.60–0.97	0.025	0.162
Epitope breadth	BLV seropositivity	154	OR 0.80	0.65–0.98	0.033	0.162
Epitope breadth	PVL	27	PVL ratio 1.26	0.88–1.82	0.211	0.316
Epitopes 1A + 2	qPCR detection	154	OR 0.44	0.15–1.29	0.135	0.237
Epitopes 1A + 2	BLV seropositivity	154	OR 0.78	0.29–2.11	0.627	0.684
Epitopes 1A + 2	PVL	27	PVL ratio 0.36	0.05–2.38	0.288	0.383

Binary outcomes were modeled by logistic regression adjusted for assay-specific daughter age and farm fixed effects. PVL was modeled using linear regression of log10(PVL), adjusted for age and farm, with HC3 heteroskedasticity-robust standard errors; effect estimates are reported as multiplicative PVL ratios. The BH-FDR family comprised all 12 analyses shown. Abbreviations and symbols: CI, confidence interval; *n*, number of daughters included in the analysis; *p*, nominal *p* value; *q*, Benjamini–Hochberg FDR-adjusted p value. “Epitopes 1A + 2” indicates predicted genotype coverage of both epitopes 1A and 2. The locus name *BoLA-DRB3* is omitted from the allele entries for brevity; the asterisk (*) is part of the allele nomenclature and does not indicate statistical significance or a footnote.

## Data Availability

The individual-level dataset supporting the analyses is provided as Supplementary Data S1. Additional supplementary tables report age distributions, maternal verification status, farm-stratified analyses, *BoLA* FDR analyses, prediction completeness, and model diagnostics.

## Supplementary Materials

The following supporting information can be downloaded at: https://www.mdpi.com/article/10.3390/ijms27198627/s1.
